# Characterization of olfactory sensory neurons in the striped ambrosia beetle *Trypodendron lineatum*

**DOI:** 10.3389/fphys.2023.1155129

**Published:** 2023-03-20

**Authors:** Twinkle Biswas, Jothi Kumar Yuvaraj, Bill S. Hansson, Christer Löfstedt, Olle Anderbrant, Martin N. Andersson

**Affiliations:** ^1^ Department of Biology, Lund University, Lund, Sweden; ^2^ Department of Evolutionary Neuroethology, Max Planck Institute for Chemical Ecology, Jena, Germany

**Keywords:** aggregation pheromone, fungal symbiont, olfactory sensory neuron (OSN), single sensillum recordings (SSR), mutualism

## Abstract

**Introduction:** The striped ambrosia beetle *Trypodendron lineatum* (Coleoptera, Curculionidae, Scolytinae) is a major forest pest in the Holarctic region. It uses an aggregation pheromone and host and non-host volatiles to locate suitable host trees, primarily stressed or dying conifer trees. The beetles bore into the xylem and inoculate spores of their obligate fungal mutualist *Phialophoropsis ferruginea* inside their excavated egg galleries, with the fungus serving as the main food source for the developing larvae. Olfactory sensory neuron (OSN) responses to pheromones and host volatiles are poorly understood in *T. lineatum* and other ambrosia beetles, and nothing is known about potential responses to fungal volatiles.

**Methods:** We screened responses of OSNs present in 170 antennal olfactory sensilla using single sensillum recordings (SSR) and 57 odor stimuli, including pheromones, host and non-host compounds, as well as volatiles produced by *P. ferruginea* and fungal symbionts of other scolytine beetles.

**Results and Discussion:** Thirteen OSN classes were characterized based on their characteristic response profiles. An OSN class responding to the aggregation pheromone lineatin was clearly the most abundant on the antennae. In addition, four OSN classes responded specifically to volatile compounds originating from the obligate fungal mutualist and three responded to non-host plant volatiles. Our data also show that *T. lineatum* has OSN classes tuned to pheromones of other bark beetles. Several OSN classes showed similar response profiles to those previously described in the sympatric bark beetle *Ips typographus*, which may reflect their shared ancestry.

## 1 Introduction

Bark- and ambrosia beetles are serious pests of conifer forests around the world. Although these two groups of beetles belong to the same family of true weevils (Curculionidae) they differ in their ecology, including host specificity, feeding behaviour and reliance on microbial symbionts (Byers, 2007). Bark beetles (Scolytinae) feed on the phloem of trees, and several species can attack and kill healthy conifers by mass-aggregation (Vasetschko, 1976; Christiansen and Bakke, 1988; Raffa et al., 2016). In contrast, ambrosia beetles (Scolytinae and Platypodinae) bore into the xylem and mainly attack dead or dying trees (Ranger et al., 2015). This contributes to nutrient recycling in forest ecosystems but may also negatively affect the forest industry when stored timber is attacked and destroyed (McLean, 1985; Wermelinger, 2004; Mueller et al., 2005; Byers, 2007).

Symbiotic relationships with fungi are essential to fulfill a variety of nutritional and ecological needs in wood-boring beetles (Hansen and Moran, 2014). In particular, the ambrosia beetles have evolved a fungus-farming lifestyle and the mutualism is obligate; the beetle larvae cannot develop in the absence of fungi (Kok et al., 1970; Beaver, 1989). The ambrosia beetles represent a polyphyletic group of beetles, presently including over 3,400 species in at least eleven lineages within the Scolytinae and Platypodinae subfamilies, which have evolved nutritional mutualism with fungi on multiple independent occasions (Farrell et al., 2001; Hulcr et al., 2007). Bark- and ambrosia beetles carry the fungal spores and thus provide the fungi with transport and host tree entrance, as well as shelter. In numerous species, the beetles also provide nutrition to fungi through specialized glandular structures called mycangia (Batra, 1963; Caldera et al., 2009; Mayers et al., 2020). In return, the fungi benefit the beetles by metabolizing host tree defenses (Blanchette et al., 1992; Kopper et al., 2004; DiGuistini et al., 2007), producing enzymes that break down indigestible wood (Valiev et al., 2009), and by providing essential nutrients such as steroids, amino acids and vitamins to beetles (Ayres et al., 2000; Bentz and Six, 2006; Bleiker and Six, 2014).

Chemical cues play a key role in the ecology of conifer-feeding beetles. These cues are detected by olfactory receptors present in the dendrites of olfactory sensory neurons (OSNs) inside sensilla on the insect antennae, each receptor with its unique response specificity (Dahanukar et al., 2005; Olsen and Wilson, 2008). Sex/aggregation pheromones are often essential for mating and aggregation on trees, host volatiles carry information of host suitability, and volatiles from non-host angiosperm trees are generally avoided (Borden, 1997; Borden et al., 1997; Zhang and Schlyter, 2004; Andersson et al., 2009). Evidence is also accumulating that odors produced by symbiotic fungi are important for the maintenance of the beetle-fungus association, which has been shown in e.g., the Eurasian spruce bark beetle, *Ips typographus* (L.), and in several species of ambrosia beetles (Hulcr et al., 2011; Kandasamy et al., 2019). For *I. typographus* the attraction towards symbiotic fungi appears to depend on the amount of volatiles released. For example, fungi that release higher amounts of 3-methyl-1-butyl acetate, 2-methyl-1-butyl acetate and 2-phenetyl acetate are highly attractive (Kandasamy et al., 2019, 2023)*.* Other studies have shown that several species of ambrosia beetles in the *Xylosandrus*, *Xyleborus*, and *Xyleborinus* genera are attracted to their fungal symbiont odors and the response may be modulated by the addition of compounds such as ethanol (Hulcr et al., 2011; Egonyu and Torto, 2018; Ranger et al., 2021). In addition, recent electrophysiological studies of the OSNs in *I. typographus* revealed several abundant OSN classes specifically tuned to odors derived from *I. typographus* fungal symbionts (Kandasamy et al., 2019, 2023). However, to our knowledge, this represents the only Scolytinae species in which detection of fungal compounds by individual OSNs has been investigated, and information from ambrosia beetles is currently lacking.

The striped ambrosia beetle, *Trypodendron lineatum* (Olivier)*,* is distributed in the Holarctic region and is considered a significant economic pest (McLean, 1985; Lindgren and Fraser, 1994). Their fungal galleries in the sapwood mainly contain the nutritional mutualist *Phialophoropsis ferruginea* (Mathiesen-Käärik) (Kirkendall et al., 2015; Lehenberger et al., 2019; Mayers et al., 2020), which is required for larval development. *Trypodendron lineatum* is attracted to the common conifer volatile α-pinene and also to ethanol that is produced in dead and dying coniferous trees in the process of anaerobic respiration as a stress response (Graham, 1968; Moeck, 1970; Vité, 1979; Borden et al., 1980; Kelsey and Joseph, 2003; Ranger et al., 2015). In addition, the female-produced aggregation pheromone (+)-lineatin (3,3,7-trimethyl-2,9-dioxatricyclononane) is central for mating and host choice (Macconnell et al., 1977), whereas several compounds from non-host trees are avoided (Roitberg and Isman, 1992; Schroeder, 1992; Zhang and Schlyter, 2004).

A pioneering study that tested common bark- and ambrosia beetle pheromone compounds, short aliphatic alcohols, vapors and extracts from host and non-host trees characterized the first OSNs in *T. lineatum* (Tømmerås and Mustaparta, 1989). Neurons responding specifically to (+)-lineatin were reported to be the most common, and neurons responding to 2-phenylethanol, ethanol or other alcohols were also identified. Yet other neurons responded to unknown compounds in the vapors or extracts of spruce, pine or birch bark (Tømmerås and Mustaparta, 1989). Since then, the knowledge of ecologically relevant compounds for *T. lineatum* has increased; in particular, the volatiles from the fungal mutualist have been identified (D. Kandasamy and M. Lehenberger, personal communication). Hence, we performed functional characterization of the OSNs of *T. lineatum*, using single sensillum recording (SSR) and a largely expanded test odor panel compared to the pioneering work by Tømmerås and Mustaparta (1989). The test odors included ecologically relevant compounds such as scolytine beetle pheromone compounds, and compounds associated with hosts, non-hosts, and fungi. We also included several compounds that are specifically detected by OSNs in *I. typographus*, the best characterized scolytine beetle in this regard (Andersson et al., 2009; Kandasamy et al., 2019; Schiebe et al., 2019; Kandasamy et al., 2023), to investigate whether sympatric bark- and ambrosia beetles may share similar OSN classes, which could be due to their common descent and/or shared odor environment of their conifer forest habitats.

We identified 13 OSN classes based on their response profiles, most of which displayed selective responses to a few structurally similar compounds. A substantial proportion of OSNs responded primarily to lineatin. The other OSN classes were responsive to pheromone compounds of other scolytine species, host or non-host compounds, or fungal-produced odors. Our results support the idea that sensing angiosperm volatiles and fungal symbionts are vital for these insects. In contrast to earlier findings (Tømmerås and Mustaparta, 1989), OSNs tuned to pheromone components of sympatric *Ips* species were also revealed, suggesting that *T. lineatum* has the ability to sense the presence of other scolytine species.

## 2 Material and methods

### 2.1 Insects

Adult *T. lineatum* individuals were collected between April and June in 2020 and 2021 from two conifer forests both located in Southern Sweden (i.e., close to Sibbhult and Långasjö, respectively), using multiple funnel traps (WitaTrap, 12 funnel size, Witasek, Austria) baited with commercial lineatin dispensers (Lineatin Kombi, Witasek, Austria). Beetles were stored alive in boxes with moistened tissue paper and kept at 4 ^°^C until the experiments.

### 2.2 Scanning electron microscopy (SEM)

SEM images of *T. lineatum* antennae were obtained to gain a better understanding of the antennal morphology and distribution of olfactory sensilla across the antennae. Five males and five females were immersed in 95% ethanol followed by critical point drying (Leica CPD 300). The dried specimens were carefully mounted onto SEM stubs using double-sided sticky tape, and sputter-coated with gold (Cesington 108 auto, 65 s, 20 mA). The preparations were viewed using a scanning electron microscope (SEM; Hitachi SU3500) at 5 kV (Microscopy Facility, Dept. Biology, Lund University).

### 2.3 Chemical stimuli

The test odor panel consisted of 57 ecologically relevant compounds, including several newly identified volatiles from the fungal symbiont *P. ferruginea* (D. Kandasamy and M. Lehenberger, personal communication; Supplementary Table S1 details the source information and purity). Other compounds were included due to their electrophysiological and/or behavioral activity in *T. lineatum* (Tømmerås and Mustaparta, 1989; Zhang and Schlyter, 2004) and other scolytine beeles, primarily *I. typographus* (Andersson et al., 2009; Kandasamy et al., 2019, 2023) and *T. domesticum* (L.) (Byers, 1992; Holighaus and Schütz, 2006), including host and non-host volatiles, volatiles from fungi associated with other scolytine beetles, and common bark beetle pheromone compounds. Stock solutions of 10 μg/μL were prepared by diluting each compound in paraffin oil. The working solutions were then prepared by further diluting the stock solutions in paraffin oil to desired test concentration. The odor stimuli (10 µL of working solution) were added to filter papers (No. 413, Whatman, VWR International) (1.5 cm × 0.6 cm), placed inside standard glass Pasteur pipettes (150 mm, VWR International) capped with a 1 mL plastic pipette tip. A pipette loaded with 10 µL paraffin oil was used as a control (blank) stimulus once per contacted sensillum. Pipettes were stored at −18°C between the experiments.

### 2.4 Single-sensillum recordings

Single-sensillum recordings were performed on adult *T. lineatum* individuals, using previously described procedures and electrophysiological equipment from Syntech GmbH (Buchenbach, Germany) (Andersson et al., 2012a; Carrasco et al., 2019). The beetles were fixed with dental wax inside a 1 cm cut section of a 200 mL pipette tip with the head and antennae protruding. The antenna was secured with dental wax on a microscope slide in a position that permitted the recording electrode to be inserted and light penetrating from below. The mounted antenna was viewed using a light microscope (Nikon, eclipse E6000FN) at ×500 magnification. Electrolytically sharpened (using 10% KNO_3_) tungsten microelectrodes were used to obtain electrical contacts with OSNs inside olfactory sensilla. The reference electrode was inserted into a pre-made hole in the pronotum and the recording electrode was inserted into the base of a sensillum. The recording electrode was connected to a piezo micromanipulator (PM 10, Märzhäuser, Wetzlar-Steindorf, Germany) for finely controlled movements. An IDAC4 (Syntech) was used to digitalize the signal, and live recordings were visualized in AutoSpike v. 3.9 (Syntech). The antenna was continuously exposed to a charcoal-filtered and humidified airflow at 1.2 L/min, which was directed onto the insect *via* a glass tube (6 mm i.d.) that terminated around 15 mm from the antenna. A 0.5 s puff (0.3 L/min; controlled using stimulus controller CS-02, Syntech) was delivered through the stimulus pipette, bringing the stimulus into the continuous airflow and onwards to the antenna. Pipettes for the initial screening of responses to the whole panel of compounds were mostly used for a maximum of two consecutive days or six odor stimulations, whereas for highly volatile compounds fresh pipettes were used for each puff to reduce effects of rapid odor headspace depletion (Andersson et al., 2012b). The dose–response pipettes were made fresh on a daily basis and used maximally for two stimulations. To characterise OSN response profiles, a high dosage of 10 μg (i.e., 10 μL solution at 1 μg/μL concentration) was utilized in the screening experiments (187 sensilla were contacted, using 48 male and 64 female individuals), and compounds were screened in random order. The OSNs were always allowed to regain their basal spontaneous activity between consecutive stimulations. OSNs were assigned to different classes based on their characteristic response profiles in the screening experiment (Table 1), and several strongly responding OSN classes were chosen for subsequent dose-response tests using additional sensilla. During the dose-response tests, the compounds were tested in increasing dosages (from 10 pg to 10 μg) to prevent sensory adaptation of the neuron, and always starting with the least active compound based on the screening results. Due to a limited number of field-caught beetles and restricted flight period, we focused our dose-response experiments on six OSN classes that primarily responded to compounds with different ecological origins, including host trees, non-host trees, the fungal mutualist, and scolytine beetles (pheromones).

**TABLE 1:** Response profile of 13 olfactory sensory neuron (OSN) classes responding strongly (≥80 Hz) to one or several compounds at the 10 μg stimulus dose.

OSN class	1	2^a^	3	4	5	6	7	8^b^	9	10^c^	11	12	13
Compound													
(±)-lineatin	+++++												
lanierone		++++						+++					
2-methyl-3-buten-2-ol			+++++			+							
(±)-2-methyl-1-butanol			++++	++++	++++						++		++
3-methyl-1-butanol			+	++++	+++++						++		++
ethanol						+							
propanol				+++									
ethyl isobutyrate				+		++++					+		+
ethyl acetate						++++							
ethyl butyrate						+++	+						+
propyl acetate				+		++++	+				+		
isopropyl acetate						+++	+						
isobutyl acetate				++	++	++					+		++
isoamyl acetate				++	++	++	+						+
2-methylbutyl acetate				++	++	+	++	+++					
6-methyl-5-hepten-2-one								++++					
(±)-1-octen-3-ol							+	+++	+++			+++++	++
(±)-3-octanol								++++	+++			+++++	++
1-hexanol						+			+			++	+++++
(*Z*)-3-hexenol									+		+	+	+++++
(*E*)-2-hexenol					+		+						++++
2-phenylethanol						+			+				+++
benzyl alcohol											++		++++
benzaldehyde											++		+++
acetophenone				++	+						+++++		+++
3,4-dimethoxytoluene										++++			
1,2-dimethoxybenzene										+++++			
styrene						+							
4-vinylanisole				++	++						+		+
4-methylanisole				++	+						+		+
2-methoxyphenol					+				+				
eugenol methyl ether											+		
terpinolene				++	++				++				+
β-pinene						++							
(+)-3-carene													+
(+)-*trans-*4-thujanol						+	+		+++++		+		+
(+)-isopinocamphone	+				+	++	++				+		+
(−)-isopinocamphone					+	++					+		
(4*S*)-*cis*-verbenol					+				+				
(−)-*trans*-verbenol						+			++				
(+)-*trans*-verbenol									++				
(−)-verbenone						+							
amitinol			++					++					++
(±)-ipsenol				+		+			++				
(±)-ipsdienol						+			+++				
geranylacetone						+	+++++						+
(±)-chalcogran			+	+								+	++
(5*S*,7*S*)-*trans*-conophthorin			++	+		+							
Neuron	A	B	A	A	A	A	A	B	A	B	A	A	A
Female n-value	22	1	7	2	0	4	1	0	1	3	0	4	1
Male n-value	15	3	2	3	2	5	0	1	3	1	2	2	5

Note: Only compounds eliciting an average response ≥20 Hz in at least one OSN, class are listed for clarity. (+) 20–39 Hz, (++) 40–59 Hz, (+++) 60–79 Hz, (++++) 80–99 Hz, (+++++) ≥100 Hz. Stimulus eliciting primary response in the 13 OSN classes, 1: (±)-lineatin, 2: lanierone, 3: 2-methyl-3-buten-2-ol, 4: (±)-2-methyl-1-butanol, 5: 3-methyl-1-butanol, 6: ethyl acetate, 7: geranylacetone, 8: 6-methyl-5-hepten-2-one, 9: (+)-*trans*-4-thujanol, 10: 1,2-dimethoxybenzene, 11: acetophenone, 12: (±)-1-octen-3-ol, 13: 1-hexanol. See Supplementary Table S2 for detailed responses in Hz.

^a^ One of the OSNs was co-localized with OSN class 1.

^b^ This OSN was co-localized with OSN class 1.

^c^ One of the OSNs was co-localized with OSN class 5, and two of the OSNs with OSN class 9.

### 2.5 Data analysis

The net responses of the neurons triggered by active compounds were calculated offline using AutoSpike v. 3.9 by counting the number of spikes (action potentials) during the initial 0.5 s of the response and subtracting the number of spikes during the 0.5 s immediate pre-stimulation period. This number was then doubled to get a response in spikes per second (Hz). Any response to the paraffin oil control was subtracted from the net odor response. At the high screening dose (10 μg), any average response below 20 Hz was considered as no response. Excitatory responses were grouped as: (+) = 20–39 Hz, (++) = 40–59 Hz, (+++) = 60–79 Hz, (++++) = 80–99 Hz, and (+++++) ≥ 100 Hz (Table 1). Recordings that could not be accurately analyzed due to poor quality contacts and data from neurons where the contact was lost before the OSNs could be confidently grouped into an OSN class were excluded from the analysis.

## 3 Results

### 3.1 Scanning electron microscopy

**FIGURE 1:**
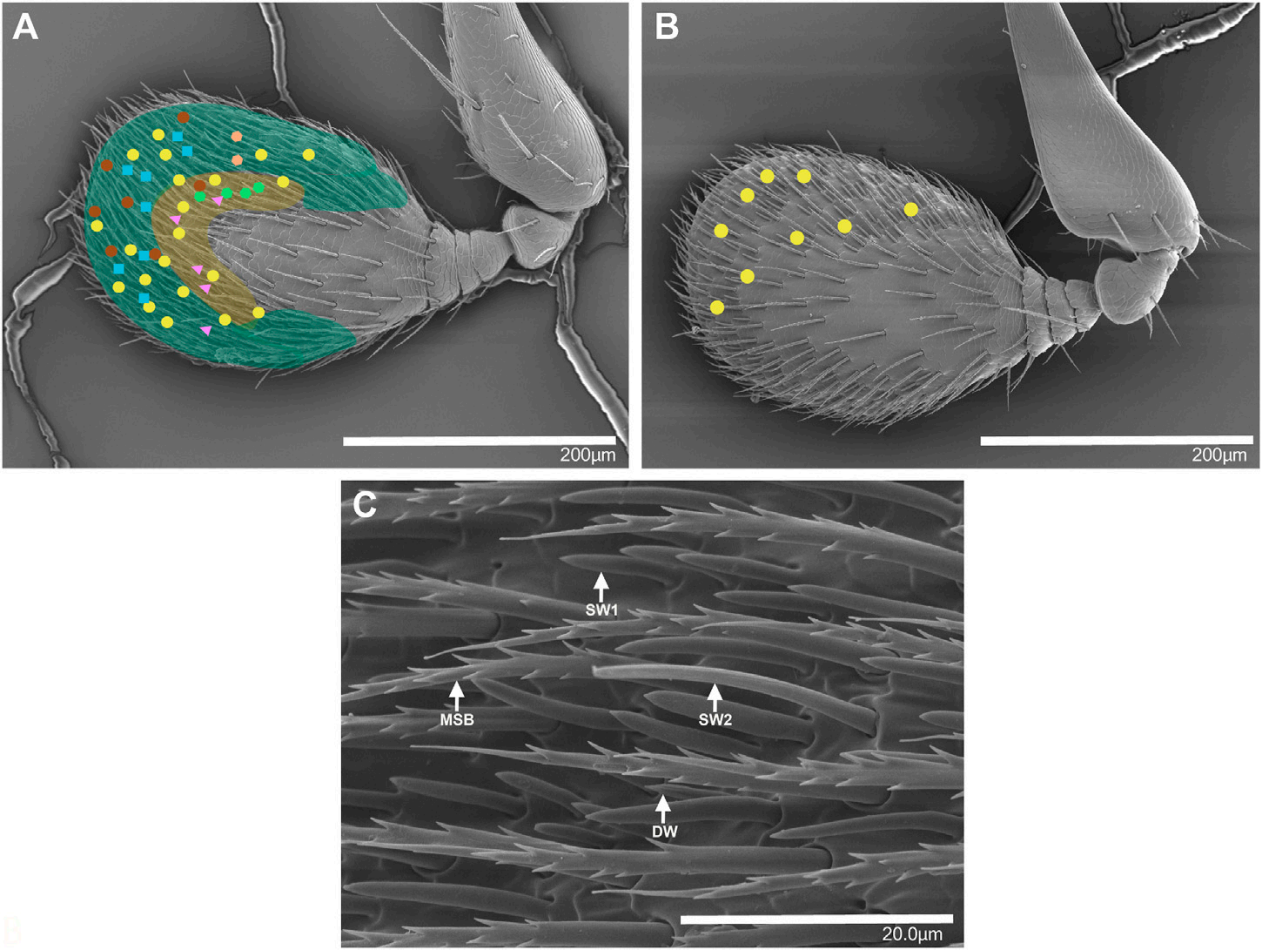
Scanning electron micrographs of the antennal club of female *T. lineatum* showing the approximate positions of six OSN classes, the antennal regions with different densities of sensilla, and different types of sensilla. **(A)** The ventral view of the terminal segment forming the antennal club. The yellow coloured area is densely covered by sensilla while the green coloured area has a lower density of sensilla. Yellow circles: lineatin OSNs (OSN class 1), blue squares: 2-methyl-3-buten-2-ol OSNs (OSN class 3), green hexagons: 2-methyl-1-butanol OSNs (OSN class 4), orange hexagons: 3-methyl-1-butanol OSNs (OSN class 5), pink triangles: ethyl acetate OSNs (OSN class 6), brown circles: (±)-1-octen-3-ol (OSN class 12). **(B)** The dorsal view of the terminal segment forming the antennal club, with additional lineatin-responsive OSNs mapped (yellow circles). **(C)** Ventral side of antennal club showing a magnified view of putative double walled sensilla (DW), putative single walled sensilla 1 (SW1), putative single walled sensilla 2 (SW2), and mechanosensory bristles (MSB).

The antennal club was approximately 280–300 μm long and 150–200 µm wide, and with no obvious morphological differences between sexes. Putative olfactory sensilla were present on both the dorsal and ventral side of the antennal club. Based on our SEM images, we could distinguish two types of putative single-walled (SW) sensilla and one putative double-walled (DW) sensillum type at various locations on the antennal club. These sensilla are similar to the sensilla in *I. typographus* for which both SEM and transmission electron microscopy images have been obtained (Hallberg, 1982). The two SW sensillum types are likely to correspond to sensillum basiconicum and sensillum trichodea type II originally described on the antennal club of *T. lineatum* (Moeck, 1968). The three types of sensilla differed in length: DW is the shortest (4–8 μm) and SW2 is the longest (20–36 μm) while SW1 is intermediate in length (10–15 μm) (Moeck, 1968). The ventral side of the antennal club is somewhat bulged and can be divided into two regions based on the abundance of sensilla (Figures 1A, C). The DW sensilla were found scattered across the antennal club and present at low density, while the central region was densely populated mainly with SW1 and SW2, with SW1 being the most abundant (Moeck, 1968). The distal and lateral regions had comparatively few sensilla overall. Mechano-sensitive bristles were also abundant on the antennal club, evenly distributed across both sides of the antennae (Figures 1A, B). The dorsal side of the antennal club is flat and has fewer sensilla than the ventral side and no obvious regions that differ in the abundance of different sensillum types.

### 3.2 General characteristics of OSN responses

**FIGURE 2:**
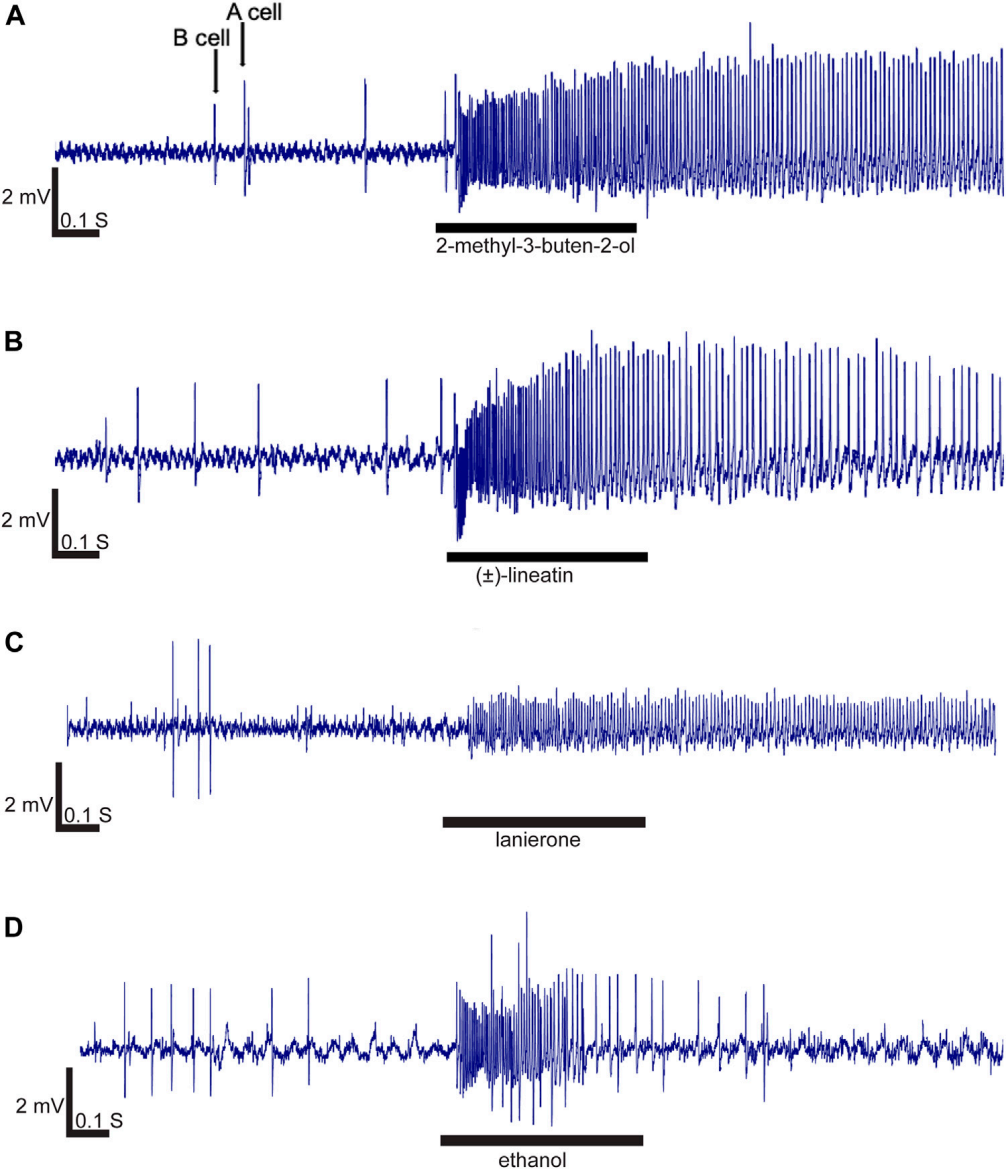
Response characteristics exhibited by four representative olfactory sensory neurons (OSNs) stimulated with 10 μg compound concentration. Excitatory responses were phasic-tonic, although some responses were more tonic than others. **(A)** Two OSNs (A and B neurons) with different spike amplitudes were generally present in the contacted sensilla. A strong and rather tonic response was elicited in the A-neuron by 2-methyl-3-buten-2-ol (OSN class 3). **(B)** Lineatin elicited a slightly less tonic A-neuron response (than in panel A) in OSN class 1. **(C)** The OSNs responding specifically to lanierone (OSN class 2) were all B-neurons. **(D)** Excitatory response evoked by ethanol showing a more phasic profile.

In total, 57 compounds were used to screen 170 olfactory sensilla of which 97 (51 female and 46 male sensilla) contained at least one OSN that responded by excitation to at least one of the compounds at the screening dosage of 10 μg. Neurons in the remaining 73 olfactory sensilla did not respond to any compound. Recordings from 17 additional sensilla were excluded from the analysis due to poor contact quality or lost contact before all diagnostic compounds had been tested, thereby preventing classification. The spontaneous spike activity of the OSNs in the absence of odor stimulation was commonly between 2 and 6 Hz. Due to such low spontaneous activity, inhibitory responses could not be used for OSN classification, and inhibitory responses were also overall rare and not repeatable. The majority of contacted sensilla contained two OSNs which could be distinguished based on spike amplitudes (large amplitude cell henceforth referred to as A neuron, and B neuron for the small amplitude cell; Figure 2A). The spike amplitudes of A and B neurons varied between the contacted sensilla (Figure 2), with the best quality contacts showing an A-neuron amplitude around 4 mV and a B-neuron amplitude around 1.5 mV (Figure 2C). Some sensilla, however, appeared to have only one OSN based on a single discernible amplitude, whereas other sensilla seemed to contain three OSNs, although the latter could not always be determined with certainty due to suboptimal signal quality. In most cases the OSNs responded by excitation to more than one compound, although only one or two compounds typically elicited the highest responses (i.e., the primary compound) and additional compounds elicited intermediate or weak secondary responses. The secondary compounds typically had similar chemical structure or the same functional group as the respective primary compound. The OSNs responded in a characteristic phasic–tonic pattern, with the maximum firing activity in the early phase of the response and subsequently declining firing until resting activity was restored (Figure 2B). The odor that provoked the largest response at the high dosage was usually also the one with the lowest OSN response threshold. The strongest responses in the OSN classes that were regarded as “strongly responding” ranged between 80 and 180 Hz.

### 3.3 Strongly responding OSN classes

Based on strong responses to one or more of the synthetic compounds at the 10 μg screening dosage, a total of 90 (female: 46, male: 44) out of 119 responsive neurons could be classified into thirteen distinct “strongly responding (response: ≥80 Hz)” OSN classes from the randomized screening (Table 1; Supplementary Table S2). Most of these OSN classes were discovered in both males and females, but some were either found only in males (OSN classes 5, 8 and 11) or in females (OSN class 7). Because only few of these OSNs were found we cannot, however, classify them as sex specific. Based on the intensity of the responses, it is likely that we have identified the key ligands for the 13 strongly responding OSN classes. A total of 33 additional OSNs from six of the strongly responding OSN classes (OSN classes 1, 3, 4, 5, 6, and 12) were exposed to dose-response tests, performed on additional sensilla after the screening experiments.

**FIGURE 3:**
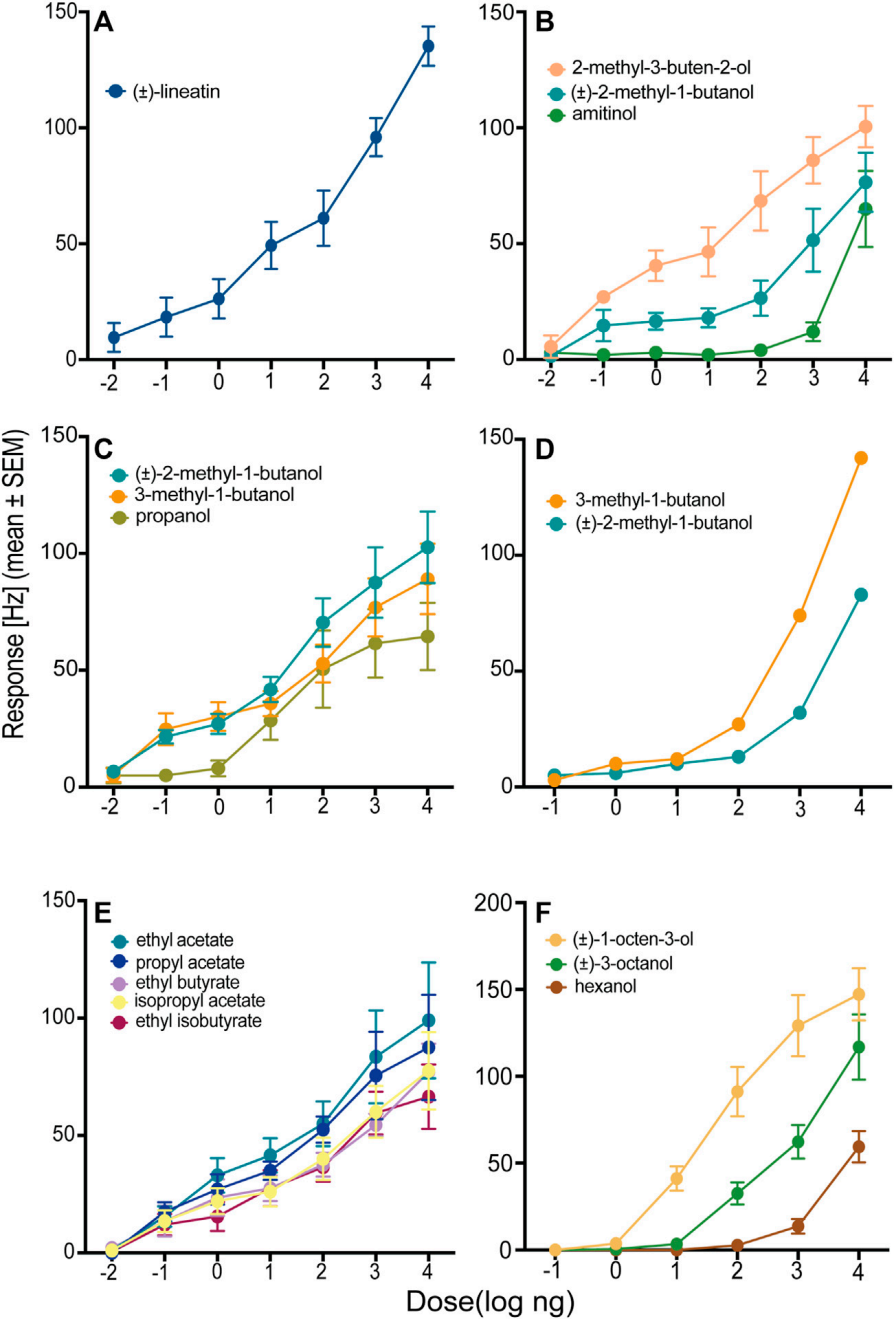
Dose–response curves of six olfactory sensory neuron (OSN) classes from *T. lineatum* with primary responses to **(A)** (±)-lineatin (OSN class 1; *n* = 8), **(B)** 2-methyl-3-buten-2-ol (OSN class 3; *n* = 4), **(C)** (±)-2-methyl-1-butanol (OSN class 4; *n* = 9), **(D)** 3-methyl-1-butanol (OSN class 5; *n* = 2), **(E)** ethyl acetate (OSN class 6; *n* = 4), and **(F)** (±)-1-octen-3-ol (OSN class 12; *n* = 7).

In total, we identified three OSN classes that responded to compounds used as attractive pheromones by different species of bark- or ambrosia beetles. Neurons responding to the *T. lineatum* aggregation pheromone lineatin (OSN class 1) were, by far, the most abundant. Lineatin-responsive OSNs were found across the entire antennal club, both on the dorsal and ventral side (Figure 1). As many as 37 of the 90 strongly responding OSNs (A cells) responded best to lineatin (Table 1; Figure 2B). Most (28) of the lineatin-responsive OSNs displayed responses to additional compounds. Although some variation among the secondary responses was observed for this OSN class, several compounds were more frequent to provoke a minor response than others. Although the average responses to the secondary compounds were weak, several individual OSNs responded by > 60 Hz, and sometimes even >80 Hz to a few of the secondary compounds (e.g., isoamyl acetate, 6-methyl-5-hepten-2-one, and 2-methylbutyl acetate; Supplementary Table S2). The response to lineatin of OSN class 1 was dose-dependent, and the response threshold around the 10 pg dose indicates high sensitivity of these pheromone-responsive neurons (Figure 3A). Thirteen of these neurons were co-localized with B neurons responding relatively weakly to other compounds (described below).

Four B neurons responded best to lanierone (2-hydroxy-4,4,6-trimethylcyclohexa-2,5-dien-1-one) (OSN class 2; Table 1 and Figure 2C), a pheromone component used by some North American *Ips* species. One of these cells was co-localized with OSN class 1, while the other three were co-localized with non-responsive A neurons. These neurons were distributed on the ventral side of the antenna.

The second most frequent class of neuron (OSN class 3, N = 9 cells) responded most strongly to 2-methyl-3-buten-2-ol (Table 1; Figure 2A), which is an aggregation pheromone compound of *I. typographus* and also produced by *I. typographus* fungal symbionts *Grosmannia penicillata* (Grosmann) and *G. europhioides* (E. F. Wright & Cain) Zipfel, Z. W. de Beer & M. J. Wingf. (Kandasamy et al., 2019). Sensilla housing this OSN class were distributed mostly at the distal part of the antennal club (Figure 1A). This neuron class also responded quite strongly to the structurally similar compound (±)-2-methyl-1-butanol and weaker to 3-methyl-1-butanol (Table 1). Secondary responses were also elicited by the spiroacetals 5*S*,7*S*-*trans*-conophthorin and (±)-chalcogran, and by amitinol. The dose-response data confirmed that OSN class 3 was highly sensitive (response threshold at 10 pg) to 2-methyl-3-buten-2-ol and that the specificity was higher at low doses, e.g., at the 100 ng dose there was no response at all to amitinol (Figure 3B).

We identified four OSN classes (OSN class 4, 5, 6 and 7) that were specifically tuned to odors produced by the fungal mutualist *P. ferruginea.* Two of these OSN classes (OSN class 4 and 5) responded quite similarly but could still be divided into two different classes based on different rank orders between the two most active compounds and variation in the secondary responses to additional compounds. Five neurons (OSN class 4) responded most strongly (mean response: 95 Hz) to (±)-2-methyl-1-butanol and these neurons also responded strongly (mean response: 92 Hz) to 3-methyl-1-butanol during the screening experiment (Table 1). Among the secondary responses, propanol triggered the most apparent (mean response: 67 Hz) response in OSN class 4 (but not in OSN class 5; described below). The dose-response tests showed stronger responses to (±)-2-methyl-1-butanol than to 3-methyl-1-butanol at the four highest doses and confirmed propanol as a secondary compound for this OSN class (Figure 3C). During screening, two neurons responded most strongly (mean response: 117 Hz) to 3-methyl-1-butanol (OSN class 5), with slightly weaker responses to (±)-2-methyl-1-butanol (mean response: 95 Hz), and clearly weaker responses to, e.g., 2-methylbutyl acetate, isoamyl acetate, and isobutyl acetate (Table 1). The dose-response experiments supported 3-methyl-1-butanol as the primary ligand for this OSN class, although the responses to (±)-2-methyl-1-butanol were rather similar at the lower doses (Figure 3D). Apart from propanol that clearly activated OSN class 4 but not OSN class 5, several of the less active compounds also differed between these OSN classes despite the relatively large overlap in response profiles (Table 1). Subsequent dose-response experiments revealed that OSN class 5 had a higher response threshold (100 pg) for its key compound 3-methyl-1-butanol as compared to the response threshold of OSN class 4 to its key compound (±)-2-methyl-1-butanol (10 pg) (Figures 3C, D). These two OSN classes were also found in different locations on the antenna: OSN class 4 neurons were mainly present in the central region of the ventral surface that is densely covered with sensilla whereas OSN class 5 neurons were present in the distal region that is less densely covered with sensilla (Figure 1A). In one of the sensilla, OSN class 5 was co-localized with a neuron belonging to OSN class 10 (described below). Nine further neurons were sensitive to several low molecular weight esters (OSN class 6), also produced by the fungal mutualist. The neurons of this OSN class were present in the ventral side around the central region of the antenna (Figure 1A). The strongest response (mean response: 92 Hz) was elicited by ethyl acetate followed by comparatively strong responses also to ethyl isobutyrate (mean response: 86 Hz) and propyl acetate (mean response: 81 Hz). We recorded intermediate responses to ethyl butyrate (mean response: 69 Hz) and isopropyl acetate (mean response: 63 Hz), and weaker responses to several additional compounds from these neurons (Table 1). Usually, the specificity of an OSN increases with decreasing doses but this OSN class responded quite similarly to the five most active compounds across all active doses (Figure 3E). One OSN (class 7) responded specifically to geranylacetone, which is also produced by the fungal mutualist, with intermediate secondary responses to (+)-isopinocamphone, 2-methylbutyl acetate and minor responses to other alcohols and esters (Table 1).

One B neuron responded strongly to 6-methyl-5-hepten-2-one (OSN class 8) produced by non-host trees, beetles as well as fungi (Table 1). This OSN class was co-localized with OSN class 1. This neuron showed secondary responses to some aromatic compounds and C8-alcohols, and it was found on the dorsal side of the antenna.

Four OSNs (OSN class 9) responded best to (+)-*trans*-4-thujanol, a compound which is produced by, e.g., fungi associated with bark beetles as well as by spruce trees. These neurons were present on both the ventral and dorsal side of the antenna. All these cells were co-localised with B neurons, two of which belonged to OSN class 10 (described below) while the other two showed only low responses and could thus not be assigned to any OSN class. The OSN class 9 showed secondary responses to several compounds, including (±)-3-octanol, (±)-1-octen-3-ol, (±)-ipsdienol, (±)-ipsenol (+)- and (−)-*trans*-verbenol, and terpinolene. Four B neurons showed best response to 1,2-dimethoxybenzene (mean response: 132 Hz) (OSN class 10; Table 1), with the structurally similar compound 3,4-dimethoxytoluene as secondary odorant also triggering a strong response (mean response: 99 Hz). These neurons were present on the ventral side of the antenna and mostly in the distal part (Figure 1A, green shaded area). The former compound has been shown to trigger strong antennal responses in the sympatric congener *T. domesticum* and is released from that species’ angiosperm host tree (*Fagus* spp.) during the host colonization phase (Holighaus and Schütz, 2006).

Two OSNs (OSN class 11) responded specifically to acetophenone—a compound that acts as an anti-attractant in several species of *Dendroctonus* bark beetles (Erbilgin et al., 2007; Erbilgin et al., 2008). Other aromatic compounds and the methyl butanols elicited clear secondary responses in these neurons (Table 1). Six neurons responded strongly to C8-alcohols emitted by angiosperm plants and fungi (OSN class 12), with strongest response to (±)-1-octen-3-ol (mean response: 126 Hz) followed by (±)-3-octanol (mean response: 110 Hz) (Figure 3F; Table 1). The mainly angiosperm-associated C6-green leaf volatile (GLV) alcohols 1-hexanol and (*Z*)*-*3-hexenol, as well as chalcogran elicited weaker responses in this neuron. Finally, we found 6 cells (OSN class 13) strongly responding mainly to GLV alcohols with primary response to 1-hexanol (mean response: 118 Hz), and clear secondary responses elicited by (*Z*)*-*3-hexenol (mean response: 103 Hz), (*E*)-2-hexenol (mean response: 90 Hz), and benzyl alcohol (mean response: 93 Hz). Several other aromatic compounds, including benzaldehyde, acetophenone and 2-phenylethanol elicited intermediate (mean response: 68–69 Hz) responses (Table 1), and additional compounds elicited yet weaker responses (Table 1). All these neurons were present on the ventral side of the antenna and were found both in the distal and the central region (Figure 1).

We found 29 (16 in females and 13 in males) neurons that only responded with weak or intermediate responses (20–79 Hz), suggesting their primary ligands were missing from the test odor panel. Hence, these neurons were not assigned to any OSN class. Twelve of these cells were A neurons and 17 were B neurons. Of the A neurons, six responded only to a single compound (isobutyl acetate, 2-methyl-3-buten-2-ol, lineatin, or 4-methylanisole, respectively), whereas the other six responded to several compounds and with different specificities (Supplementary Table S2). Among the B neurons, five responded only to isobutyl acetate (all co-localized with OSN class 1), six responded only to lanierone, two responded only to 1,2-dimethoxybenzene, one responded only to (±)-3-octanol, and one responded to isoamyl acetate and (+)-*trans*-4-thujanol, whereas two neurons responded to several compounds (Supplementary Table S2).

Finally, among the OSNs that could not be properly characterized due to lost contact before all diagnostic compounds had been tested, one OSN is worthy of mentioning as it strongly responded to ethanol (Figure 2D). Neurons responding to ethanol were previously reported for *T. lineatum* by Tømmerås and Mustaparta (1989).

## 4 Discussion

A previous electrophysiological study that mostly tested bark- and ambrosia beetle pheromone compounds and volatile compounds from plant material reported the first OSN responses in *T. lineatum* (Tømmerås and Mustaparta, 1989). Since then, the knowledge of ecologically relevant compounds for this species and other scolytines has increased dramatically. One of our main objectives was thus to identify neurons that are tuned to ecologically important compounds from a variety of biological sources, including compounds released by the fungal symbiont of *T. lineatum*. Using an expanded odor panel, we identified several novel classes of OSNs in *T. lineatum* that responded primarily to various bark- and ambrosia beetle pheromones, volatiles from host and non-host trees, or fungal-derived odors. Our findings imply that *T. lineatum* has mostly narrowly tuned OSNs, responding strongly to only one or a few structurally similar compounds and fewer broadly tuned OSNs that respond strongly to structurally non-similar compounds (Table 1). Furthermore, several of our dose–response curves indicate greater OSN specificity at lower dosages (Figure 3), which previously has been found also in other scolytines as well as in non-scolytine insect species (Larsson et al., 2001; Hallem and Carlson, 2006; Andersson et al., 2012a; Carrasco et al., 2019).

Our recordings confirm the previous findings that lineatin-responsive neurons are the most common on the antennae of *T. lineatum* (Tømmerås and Mustaparta, 1989). Whereas we only had access to racemic lineatin, the previous study showed that the lineatin-responsive neurons responded strongly to the (+)-enantiomer, which is the enantiomer that is used as the aggregation pheromone (Tømmerås and Mustaparta, 1989). Among the neurons that responded to lineatin, we observed that some OSNs responded only to this compound, whereas most of them also responded weakly, and sometimes even quite strongly, to additional secondary compounds. However, only one of these secondary compounds elicited an average response above 20 Hz at the highest tested dose (10 µg) (Table 1; Supplementary Table S2). The reason(s) for this variation among the secondary responses remains unknown but we noted that the more specific neurons were generally those with a lower maximal response to lineatin, suggesting lower overall sensitivity possibly related to beetle condition. It is possible that these neurons were simply too insensitive in order to respond to the secondary compounds. An alternative scenario could be that the lineatin-responsive neurons that also responded to additional compounds may express more than one chemoreceptor gene, which has been shown to frequently occur in mosquitos (Herre et al., 2022). However, since this has not been shown in beetles in combination with the fact that the secondary responses were clearly weaker than the lineatin response, we grouped all lineatin-responsive neurons into a single OSN class. It is unlikely that cross-contamination of stimuli during storage of pipettes in the freezer would explain the variation, because several of the secondary responses were seen also with freshly made pipettes. Olfactory sensory neurons responding to lineatin have also been found in *Thanasimus formicarius* (Cleridae), which is an important predator on ambrosia beetles (Tømmerås and Mustaparta, 1985; Tømmerås, 1988). This predator was frequently also observed in our lineatin-baited traps that we used to collect beetles for the SSR experiments.

In contrast to the previous SSR study (Tømmerås and Mustaparta, 1989), we found that *T. lineatum* also has the ability to detect aggregation pheromone components of other scolytines, including 2-methyl-3-buten-2-ol which is used by sympatric *I. typographus* and lanierone which is attractive to several species of *Ips* in North America, including *I. pini*, *I. avulsus*, and *I. integer* (Miller et al., 1997; Erbilgin et al., 2003; Miller et al., 2005; Birgersson et al., 2012). The specific detection of 2-methyl-3-buten-2-ol is interesting as it is used by a sympatric aggressive bark beetle that is able to kill healthy trees. The ecological relevance of 2-methyl-3-buten-2-ol for *T. lineatum* is however unknown, but it may be used to locate a potential host tree. Indeed, based on captures of *T. lineatum* in traps baited with *I. typographus* pheromone compounds, it has been hypothesized (Benz et al., 1986; Borden et al., 1997) that *T. lineatum* may use these compounds to find a tree killed by the bark beetle, as *T. lineatum* does not kill trees themselves. Even if *I. typographus* has attacked a tree at the same time, *T. lineatum* should be able to locate enough uninfested host material to bore into the xylem and make their fungal galleries without any obvious competition because *I. typographus* lives under the bark in the phloem. However, over the course of our own field trapping experiments with *I. typographus*, no significant by-catches of *T. lineatum* have been observed (M.N. Andersson, personal observation), suggesting this hypothesis remains to be tested. Moreover, the detection of lanierone by another dedicated OSN class provides further evidence that *T. lineatum* has the capacity to detect interspecific signals. Such signals may play important roles in the interactions between sympatric bark- and ambrosia beetles, and in the case of lanierone, perhaps the North American *Ips* species that produce this compound.

Recent studies revealed that the bark beetle *I. typographus* possesses several OSN classes that primarily respond to volatiles produced by its various fungal symbionts (Kandasamy et al., 2019; Kandasamy et al., 2023). Compared to these symbionts, the fungal mutualist (*P. ferruginea*) of *T. lineatum* produces a different bouquet of volatiles, and we here showed that the beetle indeed has the ability to detect these volatiles. The fact that a large proportion of neurons were specifically activated by fungal compounds implies that the odors from this obligatory mutualist are important for the beetles. Four different OSN classes (OSN class 4, 5, 6 and 7) appear to be main responsible for this interaction, and two of them have evolved different specificities for two structurally similar compounds, 2-methyl-1-butanol (class 4) and 3-methyl-1-butanol (class 5), respectively. The third OSN class (class 6) showed a more indiscriminate response to several low molecular weight acetate and butyrate esters, although it should be noted that we did not have the possibility to correct for differences in compound release rates from the odor cartridges (Andersson et al., 2012b). The fourth OSN class (class 7) responded rather specifically to geranylacetone with secondary compounds being clearly less active. Collectively, these four OSN classes responded to nearly all the compounds produced by the fungal mutualist (Supplementary Table S1). It has been demonstrated in another species of ambrosia beetle, *Xylosandrus germanus* (Blandford)*,* that it can differentiate between fungal species through their volatile profiles (Ranger et al., 2021). Whether *T. lineatum* can discriminate between different fungal species and whether it is attracted to the specific odor blend produced by *P. ferruginea* remain to be investigated in future studies.

Host-produced monoterpenes and ethanol have been shown play a role in host selection by *T. lineatum* (Vité, 1979; Borden et al., 1980). Surprisingly, we only characterized one OSN class (9) primarily responding to a monoterpenoid compound from host trees, (+)-*trans*-4-thujanol (which is also produced by fungal symbionts of *I. typographus*) (Blažytė-Čereškienė et al., 2016; Kandasamy et al., 2023). Several neurons responding to ethanol were reported in a previous study (Tømmerås and Mustaparta, 1989), but we found only one neuron strongly responding to ethanol. Unfortunately, the contact was lost before the complete odor panel had been tested (this neuron is therefore not shown in Table 1; the response is shown in Figure 2D), but it is likely that this neuron correspond to the ethanol-specific OSN class previously reported.

Several studies have shown that ambrosia beetle host selection behavior is influenced by non-host volatiles, however different species respond to different but frequently overlapping sets of compounds (Borden et al., 1997; Deglow and Borden, 1998; Borden et al., 2001; Zhang and Schlyter, 2004; Pham et al., 2020). In *Trypodendron* spp. it is known that a number of non-host volatile compounds such as 1-hexanol, (*E*)-2-hexen-1-ol, (*Z*)-2-hexen-1-ol, (*Z*)-3-hexen-1-ol, and (±)-1-octen-3-ol reduce positive responses to pheromones and even host volatiles (Nijholt and Schonherr, 1976; Borden et al., 1997; Campbell and Borden, 2009). Here we found that OSN class 13 responded primarily to 1-hexanol and (*Z*)-3-hexen-1-ol followed by (*E*)-2-hexen-1-ol and several aromatic compounds, whereas OSN class 12 primarily responded to (±)-1-octen-3-ol followed by (±)-3-octanol. Tømmerås and Mustaparta (1989) also found that some receptor neurons in *T. lineatum* responded exclusively to bark vapors from non-host birch trees, but the active compounds were not identified. Finally, we observed strong OSN responses to 1,2-dimethoxybenzene and the structurally similar compound 3,4-dimethoxytoluene in OSN class 10. The former compound is released by beech (*Fagus sylvatica*) trees during the decay phase that is attractive to the sympatric congener *T. domesticum*, and it elicits strong antennal responses in that species (Holighaus and Schütz, 2006). The ability to detect this compound is thus conserved across these two species, and the compound may act as an angiosperm non-host cue for the conifer specialist *T. lineatum.*

Of all species in the Scolytinae family, the bark beetle *I. typographus* is arguably the best characterized species in terms of OSN responses (Andersson et al., 2009; Kandasamy et al., 2019; Schiebe et al., 2019; Kandasamy et al., 2023). Comparing the OSN response profiles in *T. lineatum* with those in this bark beetle, we found that five OSN classes share the same key ligand in these two species (Andersson et al., 2009; Kandasamy et al., 2019; Schiebe et al., 2019). Specifically, the OSN classes primarily tuned to (±)-1-octen-3-ol (OSN class 12 in *T. lineatum* and OSN class C8en in *I. typographus*) and to 2-methyl-3-buten-2-ol (OSN class 3 in *T. lineatum* and OSN class MB in *I. typographus*) have highly similar response profiles in the two species, although there is some modest variation among the less active ligands (Andersson et al., 2009; Kandasamy et al., 2019). Moreover, in three other OSN classes the most active compounds are the same in both species, but these neurons differed in their secondary responses to a larger extent (20–79 Hz). Specifically, the OSN classes with primary responses to geranylacetone, (+)-*trans*-4-thujanol, and C6-alcohols (OSN classes 7, 9, 13 in *T. lineatum* and OSN classes tMTol, GA, and GLV-OH in *I. typographus*; Andersson et al., 2009; Schiebe et al., 2019; Kandasamy et al., 2019) are all more broadly tuned in *T. lineatum* compared to *I. typographus* with additional compounds eliciting secondary responses and also stronger responses. This is especially the case for OSN class 13 in *T. lineatum* for which comparatively strong secondary responses (68–93 Hz) were found for benzyl alcohol, benzaldehyde, 2-phenylethanol and acetophenone. Responses to these compounds in the GLV-OH OSN class in *I. typographus* are less pronounced (Kandasamy et al., 2019).

Previous comparisons of OSN response profiles across other beetle species that are closely related to each other revealed a high degree of functional conservation. For example, when the beetles *Pachnoda marginata* (Drury) and *P. interrupta* (Olivier) (Scarabaeidae) were confronted with food-associated scents they showed similar OSN responses despite the fact that they occupy different habitats (Bengtsson et al., 2011). Similarly, OSN response conservation has also been seen in the clover seed weevils *Protapion fulvipes* (Geffroy) and *P. trifolii* (L.), with eight reported OSN classes displaying highly similar responses in both species (Andersson et al., 2012a; Carrasco et al., 2019). It is possible that the responses of functionally conserved OSN classes are mediated *via* conserved odorant receptors (ORs) in closely related species. For example, several single-copy (1:1) OR orthologues have been identified in different species of the Curculionidae family (Roberts et al., 2022). In that study, two sets of such OR orthologues were functionally characterized in three species, the bark beetles *I. typographus* and *Dendroctonus ponderosae* (Hopkins) as well as the pine weevil *Hylobius abietis* (L.)*.* One set of orthologues responded similarly to several C6-alcohols, and the other set responded exclusively to 2-phenylethanol (Roberts et al., 2022). It remains to be investigated whether the OSN classes characterized here with similar responses as OSN classes in *I. typographus* express ORs that are conserved across the Scolytinae subfamily.

Our SEM analysis of the *T. lineatum* antennae showed that single-walled sensilla are the dominant type of olfactory sensilla, and our electrophysiological recordings were performed on these sensilla, although we could not discriminate between SW1 and SW2 in the light microscope. Similar to *I. typographus* (Andersson et al., 2009), both sexes appear to have similar abundances of the different OSN classes and similar antennal morphology (see also Moeck (1968) for a detailed comparison between sexes), suggesting that males and females detect the same compounds; however, whether they respond the same behaviorally remains unknown. Also similar to *I. typographus*, we found that several of the OSN classes were not randomly distributed across the antennae (Andersson et al., 2009; Kandasamy et al., 2019). For example, OSNs responding to 2-methyl-3-buten-2-ol (OSN class 3) and 3-methyl-1-butanol (OSN class 5) were distributed in the distal region (Figure 1, green shaded area), whereas OSNs responding to 2-methyl-1-butanol (OSN class 4), low molecular weight esters (OSN class 6), and C6-green leaf volatile alcohols (OSN class 13) were found only in the central region of the antenna (Figure 1). In contrast, other OSN classes such as OSN class 1 that respond to lineatin were found across the entire antennal club. However, it should be noted that OSN classes for which we only found few neurons (e.g., OSN class 5) may have wider spatial distributions across the antennae than observed here.

In conclusion, we report 13 functionally characterized and strongly responding OSN classes in *T. lineatum*. We discovered that, in addition to highly abundant OSNs that respond to the aggregation pheromone lineatin, *T. lineatum* has several OSN classes that respond to host, non-host, and fungal-derived odors and others are tuned to bark beetle pheromone compounds. Strikingly, our results suggest that *T. lineatum* has four OSN classes that together detect essentially all volatiles produced by their fungal mutualist *P. ferruginea*, which indicates that fungal odors may be important in the maintenance of this association*.* A comparison between *T. lineatum* and *I. typographus* reveals that some of the OSN classes that respond to volatiles emitted by the host or non-host plants, or beetles (pheromones) respond similarly in the two species, suggesting that some olfactory functions are conserved within the Scolytinae subfamily. Several neurons did not respond strongly to any compound in our odor panel which implies that our experiments did not include all compounds that are relevant to this beetle. Although further research is needed to fully comprehend the chemical ecology of *T. lineatum*, this work adds one piece to the puzzle, demonstrating the utility of the SSR approach in screening for chemicals with biological activity. On this note, the newly discovered physiologically active compounds should be tested for behavioral activity, not the least for the development of improved semiochemical-based management of this pest insect.

## Data Availability

The original contributions presented in the study are included in the article/Supplementary Material, further inquiries can be directed to the corresponding author.

## Supplementary material

SUPPLEMENTARY TABLE S1: Test compounds with information of source, purity, and examples of biological origins.

SUPPLEMENTARY TABLE S2: Action potential responses (Hz) of the individual neurons classified into 13 strongly responding olfactory sensory neuron (OSN) classes at the 10 μg screening dose, as well as responses of neurons not classified due to absence of strong (≥80 Hz) responses.
